# Investigation on the occurrence of *Echinococcus multilocularis *in Central Italy

**DOI:** 10.1186/1746-6148-5-44

**Published:** 2009-11-26

**Authors:** Pietro Calderini, Marta Magi, Simona Gabrielli, Alberto Brozzi, Susanna Kumlien, Goffredo Grifoni, Albertina Iori, Gabriella Cancrini

**Affiliations:** 1 Istituto Zooprofilattico Sperimentale delle Regioni Lazio e Toscana, Sezione di Rieti, Via Tancia 21, 02100 Rieti; 2 Dipartimento di Patologia Animale, Profilassi ed Igiene degli Alimenti, Università di Pisa, Viale delle Piagge 2, 567124 Pisa, Italy; 3 Dipartimento di Scienze di Sanità Pubblica, "Sapienza" Università di Roma, Piazzale Aldo Moro 5, 00185 Roma, Italy; 4 Istituto Zooprofilattico Sperimentale delle Regioni Lazio e Toscana, Sezione di Viterbo, Strada Terme, 01100 Viterbo, Italy

## Abstract

**Background:**

Recent studies on geographic distribution of *Echinococcus multilocularis *in Europe show that it has a wider range than previously thought. It is unclear, however, if the wider distribution is due to its recent spreading or to a lack of previous data from the new areas. Italy, previously considered *E. multilocularis*-free, is now part of these new areas: infected foxes (the main definitive host of the tapeworm) have been observed in a Northern Alpine territory. Thus, more surveys need to be done in other Italian regions in order to monitor the spreading of *E. multilocularis*. The aim of the present study was to look for this parasite in 283 foxes collected in an Apennine area of Central Italy by different diagnostic methods.

**Results:**

The foxes were heavily parasitized by 11 helminthic genera, but none of the animals was infected by *E. multilocularis *neither by *E. granulosus *(harboured adult worms or their DNA). Low specificity was observed in commercially available ELISA kits for the detection of *E. multilocularis *antigens in the faeces. Molecular diagnostics were sensitive and specific for the detection and identification of tapeworm eggs in faeces, but less sensitive, although specific, to adult tapeworms in the intestinal content.

**Conclusion:**

Preliminarily, we can say that no *E. multilocularis *could be found in the study area. These data will enable us to follow temporal changes of the spatial distribution of the parasite in the study area of the Central Apennines. Due to its low specificity the ELISA kit for *E. multilocularis *coproantigens is not suitable for epidemiological surveys, whereas molecular diagnostics applied to faecal samples give useful results. Finally, absence of *E. granulosus *in foxes living in the endemic areas studied confirms the thought that this tapeworm prefers a different definitive host.

## Background

Alveolar hydatidosis due to infection with the larval stage of *Echinococcus multilocularis *is one of the world major zoonoses [1]. *E. multilocularis *depends on its definitive and intermediate hosts, which harbour the parasite at intestinal level and in internal organs, respectively. In various endemic regions, different definitive and intermediate hosts may be involved in the transmission cycles, but the most important definitive host is the red fox (*Vulpes vulpes*), whereas several small rodents (mainly Arvicolidae and Cricetidae) may act as intermediate hosts. Therefore, transmission of *E. multilocularis *usually occurs in a sylvatic cycle, which is sometimes linked, via infected small mammals, to domestic dogs and cats. The infected fox sheds viable eggs of *E. multilocularis *with his faeces, thus contaminating the food of small rodents and infecting them. A fox devouring the parasitized rodents then closes the cycle. Man is an accidental host; infection takes place by swallowing infectious eggs present on possibly contaminated materials and produces, as in all intermediate hosts, tissue alveolar hydatidosis, a potentially lethal disease.

*E. multilocularis *is widespread all over the Northern hemisphere. The wide distribution of foxes and the ubiquity of susceptible small mammals in most European habitats suggest that virtually all regions might be suitable for *E. multilocularis *transmission; however, in some European regions the parasite has so far not been recorded. Now, reports on a growing number of areas where the parasite has been observed [2], a raise of prevalence of infected animals [3,4], an increasing fox population [5], the establishment of the parasite cycle in urban areas [6] and, finally, a growing number of alveolar hydatidosis in humans and other accidental hosts [1,7] put some questions on the real distribution area of the parasite.

In Europe, the parasite has been observed in several countries, especially in and around the Alps and in the European part of Turkey [8-11]; Italy has been considered *E. multilocularis*-free until 2001. Former surveys performed in the Alpine regions [12-14] suggested that Alpine mountains may act as an insurmountable (mechanical, climatic, ecological) barrier for the parasite spreading [15]. Then the worm was observed for the first time in red foxes of the province of Bolzano, the Northernmost part of the country, and its presence was subsequently confirmed by several studies in the same geographical area [15-18]. Here, its relatively high prevalence suggests that the parasite has an autochthonous life cycle, hypothesis supported by the results of recent genetic studies that recognized the Italian isolates as a cluster of *E. multilocularis *genotypes, unique if compared to the genotypes from Europe and Alaska [15]. Therefore, its presence in the area is probably not a result of the recent immigration of infected foxes from surrounding regions, but it is an ancient presence, very slowly consolidated and only now detected. Thus, more surveys need to be done in other Italian regions in order to monitor the existence of *E. multilocularis *and its strains, and to define its possible intermediate hosts. The study area has to have a high density of suitable hosts (mainly foxes and rodents) and be in a mountainous region, as shown in the Alpine area where the parasite is abundant. Furthermore, the study area has to be at some distance from the province of Bolzano in order to fix the distribution limits of the parasite. The Central Apennines turned out to be an appropriate site to start our survey.

The second aim of the present study was to evaluate the efficacy of innovative laboratory techniques for the rapid examination of fox samples. The quick screening of a high number of samples is particularly helpful in areas such as the one under study, where the prevalence of the pathogen is expected to be low or even zero. High levels of sensitivity and specificity of the parasitological test are relevant for an accurate epidemiological definition of the study area [19].

Finally, a collateral aim of the study was to evaluate the role of the fox as definitive host for *E. granulosus*, in areas where cystic hydatidosis heavily affects the sheep.

## Results

### 1. Macro- and microscopical examination

After the macroscopic inspection and after the examination with the stereomicroscope of the intestine content of 283 foxes, we found 237 positive subjects (83.7%), in which we could identify 11 helminthic genera, often in association: *Taenia, Dipylidium, Mesocestoides, Toxocara, Toxascaris, Trichuris, Capillaria, Ancylostoma, Uncinaria*, and *Opisthorchis*. Further examinations of all the results are in progress, however, we can assess that, as for cestodes found in 134 foxes collected in Tuscany (the Northernmost part of the study area), *Dipylidium *spp. was the most prevalent parasite (57%), occurred with high mean intensity (80) and was the dominant species (*I *= 58%). *Mesocestoides *spp., the second most frequent genus, had a prevalence of 44.6% and was codominant (*I *= 38%). Their prevalence and density were higher in mountain areas than in other considered environments. Only twelve foxes had *T. taeniaeformis *(4.2%). No sample containing *E. multilocularis *neither *E. granulosus *could be found.

By light-microscopy of faeces in fresh state, concentrated by flotation, and concentrated by sedimentation we could identify cestode eggs in 6/283 animals (2%). This finding could not give a definitive answer on the presence of *E. multilocularis/E. granulosus*, since their eggs are morphologically indistinguishable from other cestode eggs.

### 2. Coproantigen

After the screening of 166 faeces by the ELISA kit Chekit Echinotest Monophasic^® ^38 samples were positive and 11 were ambiguous. The re-examination of the same 166 faeces samples with the ELISA kit Chekit Echinotest Biphasic^® ^gave 26 positive and 3 ambiguous results. Comparing the two kits the positive results of the monophasic kit, i.e. 38 samples, were confirmed in the biphasic kit by 13 samples and 1 sample was ambiguous. Out of the 11 ambiguous samples at the first test, 3 turned out to be positive and 8 negative at the second test. Furthermore, 34 positives at the first test were negative at the second, and 14 negatives at the first test were positive at the second. If we consider positive the ambiguous results, the two tests may be compared as reported in table 1. The two tests concord in 71.1% of the results. Following the evaluation table of Altmann the index K is 0.21 (L.C. 95%; I.C. 0.036-0.38) that means the results are fairly discrepant.

**Table 1:** Synthesis of the results drawn by the application of the ELISAs Chekit Echinotest monophasic and biphasic.

	biphasic positive	biphasic negative	Total
monophasic positive	15	34	49

monophasic negative	14	103	117

total	29	137	166

All foxes positive to the Echinotest harboured at least tapeworms.

### 3. Molecular analyses

**Figure 1:**
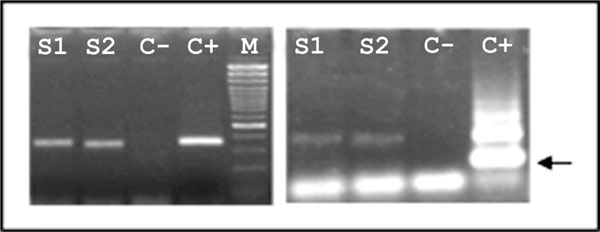
**On the left: PCR of the mitochondrial gene 12S rDNA tested on 2 samples (S1 and S2) positive for cestodes; negative (C-) and positive to *E. multilocularis *(C+) controls, and molecular weight indicator of 100 bp (M)**. On the right: nested PCR of the same samples (S1 and S2) and of the control samples (C-, C+ to *E. multilocularis*). The arrow indicates the diagnostic band.

After screening by PCR for the identification of at least 12 species of cestodes we found that out of 184 intestinal contents 30 were positive (16.3%) and out of 232 faeces 16 were positive (6.9%). These positive samples were tested again by the nested PCR, which did not identify any sample positive to *E. multilocularis *or *E. granulosus*. The sensitivity of these analyses, evaluated on samples positive to detectable tapeworms (i.e. *Mesocestoides *and *Taenia*), was 22.8% (18/79). The amplicons obtained at the first and at the second PCR from samples of intestinal content are shown in the Figure 1. In the first photograph the positive samples are on the application sites S1 and S2, on their right is the positive control for *E. multilocularis *(C+). In the second photograph the nested PCR shows the characteristic band (250 bp) of *E. multilocularis *on the application site of the positive control only.

As identified in the morphological examination, the sequencing of the amplicons confirmed the presence of parasites of the genera *Mesocestoides *(identity ranging from 87% and 92% to *M. lineatus *DNA, accession number L49450) and of *T. taeniaeformis *(identity ranging from 98% and 99%, accession number EU219537) explaining thus the positive results of the first PCR.

## Discussion

Alveolar hydatidosis is an emerging zoonosis due to a parasite whose life cycle is still not completely defined, as is its real geographic distribution. The increase of its distribution area in Europe now registered [8] could be due to its spreading into previously parasite-free areas or to a previous lack of information from these areas. Italy is one of these newly positive countries, where the finding of the parasite in an Eastern Alpine region raised the question whether an autochthonous cycle exists in Italy or whether the focus evidenced originated from the neighbouring countries. Multi-locus microsatellite analysis supports the hypothesis of the presence in Italy of an ancient autochthonous cycle of *E. multilocularis *[15], and justifies further surveys to characterize the entity of this focus and its current spatial limits.

The survey we started was in an area at some distance from the positive province, where climatic conditions are favourable to the dispersal and the viability of the eggs and where susceptible small rodents are present [20]. In view of the wide distribution of the foxes, the Apenninic area might be suitable for the parasite transmission. However, *E. multilocularis *was not found, although we detected *T. taeniaeformis*, which shares the same transmission route (fox-rodent), and *M. lineatus*, which includes rodents among its intermediate hosts.

The fox sample size might have hampered the detection of positive animals, since the distribution of parasite numbers in the fox population is rather heterogeneous, with a small number of foxes carrying most of the parasite biomass.

Concerning the diagnosis, at present the reference method to detect *E. multilocularis *in the fox is the removal of the intestine during necropsy and the application of the sedimentation and counting technique (SCT), which has a 100% sensitivity and specificity [10]. However, this method is laborious and time-consuming, thus actually not suitable for epidemiological surveys. For this reason, techniques that simplify the searching for *E. multilocularis *in the fox have been developed, as the identification of amplified DNA or the screening for antigens in the faeces. The first technique has a 100% specificity [19] as confirmed by the present study, but shows an 89% sensitivity [21] or even less [22]. In this survey the considerable quantity of mixed helminthic species often present in the same intestinal content could have affected the equal distribution of the material (each sample was divided in aliquots for all the examinations), hampering thus the performance of the first PCR in the detection of *M. lineatus *and *T. taeniaeformis *DNA. Nevertheless, this technique is especially appropriate for the screening of areas where the prevalence is unknown and it is particularly indicated for the identification of cestode eggs in faeces, which may also be collected from the ground, when fox carcasses are not available. On this sample molecular diagnostics allowed the identification of each tapeworm and was more sensitive than the concentration method (7% versus 2%).

The ELISA test for the identification of *E. multilocularis *coproantigens is scarcely sensitive and specific [23,25]. Since no positive specimen for *E. multilocularis *has been found in the present study, an evaluation of the test sensitivity could not be done. The biphasic ELISA test showed some improvement in the specificity if compared to the monophasic ELISA, but not enough to take this test in consideration.

As a collateral result of the survey, since *E. granulosus *in these regions is present (it is reported in about 50% of the sheep regularly slaughtered), our data confirm the thought that this tapeworm prefers a different definitive host.

## Conclusion

None of the 283 foxes examined during the years 2004-2007 was infected by *E. multilocularis *(had adult worms or its DNA), and this enables a subsequent survey in the same area to assess a possible spreading of the parasite from other areas. However, further investigations on the small rodents that act as intermediate host of the Italian *E. multilocularis *isolates are necessary, and future surveys need to be done to investigate whether *E. multilocularis *will still be absent in the Southern part of Europe or whether it is spreading southwards. In fact, considering the epidemiological evidences that suggest that migration or dislocation of infected hosts can play a role in the parasite spread [1], the absence of *E. multilocularis *in the foxes examined in the present study does not exclude definitively its presence in areas where the ecological and environmental conditions are favourable to its transmission. On the contrary, the absence of *E. granulosus*, confirms that the fox doesn't seem one of the preferred definitive hosts of this parasite.

Finally, we can assess that the low specificity shown by the ELISA kit make this test not suitable for epidemiological surveys, especially in areas like that screened in the present study, where the expected prevalence is low or zero. In this case molecular diagnostics applied to faecal samples produce useful results.

## Methods

### 1. Host and study area

The study was focused on the fox. Foxes examined were shot by hunters (according to the regional Law 157/92 and regulations, during a control program of the fox population), or were found dead (because of road traffic accident or poisoning) and delivered to the Istituto Zooprofilattico del Lazio e della Toscana and to the Department of Animal Pathology of Pisa University for pathological analyses. Ethical approval was not needed for the study.

During the years 2004-2007 a total of 283 carcasses were conferred on and submitted to the institutional controls and to parasitological analyses. All animals were collected in the Central Apennines (regions Tuscany and Latium), in areas at 0-1200 m above sea level, 42.3°-43.5°N and 11°-13°E. Climate is classified as apenninic-continental, with severe-cold-temperate winters, warm summers, variable rainfall, snowfall. The study area includes mainly highland and midland but also lowland habitats. It is a complex mosaic of environments (forest, grassland, moorland, woods, deep valleys, streams, lakes, marshes, peat land, cultivated lands, human settlements) that hold an extremely wealthy biodiversity. The wild fauna includes small rodents possible intermediate hosts of the parasite (as *Arvicola terrestris*, *Microtus savii, Mus musculus*, and *Clethrionomys glareolus*) [20].

The carcasses conferred on where stored at -20°C, and for the examinations thawed at room temperature for 48 hours.

### 2. Organ removal and storage

In order to prevent the spill of the intestine content during its removal two tight knots about 5 cm from one another were made under the pyloric region and the same was done under the rectum. A cut between the two knots under the pylorus and a cut between the two knots at the rectal extremity separated the intestine from the carcass. The portion was then frozen at -80°C for about 5 days, thawed, separated from the omentum, distended and cut in segments of about 15 cm. Each segment was then cut longitudinally and put in a conical glass containing water. The content of each segment was carefully separated from the intestine wall under water and was allowed to sediment for 30 minutes [10,26]. Then, it was shared in two aliquots, for microscopy and molecular diagnostics, like the material obtained from the intestinal wall scrapings.

Faeces were removed from the rectum and stored in three aliquots for three different examinations: microscopy, searching for coproantigens, and DNA identification by PCR. All aliquots were frozen at -20°C and the samples for PCR were put in 70% ethanol.

### 3. Macro and microscopical examination

After opening the intestine segment, a macroscopical examination of the material was performed to screen for large parasites. After sedimentation of the intestine content the supernatant was removed and the whole sediment split into about 15 aliquots in 15 Petri dishes and examined by means of a stereomicroscope. The parasites found were counted, and their taxonomic position was determined following the guidelines of Levine [27] and Soulsby [28].

The faeces samples were then examined again, this time by means of a light-microscope at high magnification, after the applying of the classic concentration techniques (flotation and sedimentation).

### 4. Coproantigen

For the identification of *E. multilocularis *coproantigen from faeces samples we used the ELISA kit of Bommeli Diagnostic (Chekit Echinotest Monophasic^®^) taking for each animal 1 g of the sample following the instructions of the producer. The percentage of the optical density (OD) was given by the rate: OD sample-OD negative control/OD positive control-OD negative control. Samples showing a percentage inferior to 30 were considered negative, those ranging between 30% and 40% ambiguous, and samples above 40% positive.

The same samples were re-examined with the kit produced successively (Chekit Echinotest Biphasic^®^) by the same producer. With both test systems we screened 166 individuals out of the 283 collected.

### 5. Molecular analyses

The DNA searching for *E. multilocularis *by PCR was performed on samples of faeces (n = 232) and samples of scraped materials and intestinal contents (n = 184), i.e. part of the intricate mass of the worms. The DNA from the faeces was extracted using a kit produced by QIAGEN (QIamp DNA Stool^®^, Milano), whereas that from the intestine content was extracted using a kit produced by Promega (Wizard SV Genomic DNA Purification Kit^®^, USA).

The extracted DNA was amplified following a two-steps path that identifies a region of the mitochondrial gene 12S rDNA of *E. multilocularis *[21]. In the first step, primers P60.for. and P375.rev. amplify the fragment 373 bp shared by at least 12 species of cestodes: *E. multilocularis*, *E. granulosus, Taenia hydatigena, T. martis, T. taeniaeformis, T. crassiceps, T. mustelae, T. ovis, T. pisiformis, T. polyacantha*, *T. serialis*, and *Mesocestoides leptothylacu*s,. In the second step the amplification products were subjected to a couple of primers Pnest.for. and Pnest.rev. in a nested PCR that amplifies a fragment of 250 bp specific for *E. multilocularis*. Positive and negative controls were run in all amplification procedures.

Samples proven positive at the first PCR and negative at the second PCR specific for *E. multilocularis *were examined further for a stricter identification based on their genetic identity with cestode species already studied and available in GenBank. The amplicons produced in the first PCR were removed from the gel, purified and sequenced (MWG-Biotech, Germany). The sequences obtained were assembled, corrected by visual analysis of the electropherogram using Bioedit v.7.0.2 [29] and subjected to Blast identity search (NCBI) to give the most likely identification.

## Authors' contributions

GC conceived of the study, participated in its design and helped to draft the manuscript. PC has made substantial contribution to conception, design and coordination of the study. MM carried out necropsies, parasitological and immunological analyses on foxes from Tuscany. SG carried out the molecular genetic studies. AB participated in the parasitological and immunological analyses on foxes from Latium and in the interpretation of the results. SK participated in the parasitological and immunological analyses on foxes from Latium and drafted the manuscript. GG carried out necropsies and parasitological analyses on foxes from Latium. AI carried out the morphological identification of intestinal helminths collected. All authors read and approved the final manuscript.
